# Genotoxicity Induced by Carcinogenic Agents or Occupational Exposure with Sufficient Evidence for Bladder Cancer

**DOI:** 10.3390/jcm14134492

**Published:** 2025-06-25

**Authors:** Edyta Kasperczyk, Kateryna Tarhonska, Ewa Jablonska

**Affiliations:** 1 Department of Translational Research, Nofer Institute of Occupational Medicine, 91-348 Lodz, Poland; kateryna.tarhonska@imp.lodz.pl; 2 Laboratory of Molecular Markers and Biostatistics, Department of Chemical Safety, Nofer Institute of Occupational Medicine, 91-348 Lodz, Poland; ewa.jablonska@imp.lodz.pl

**Keywords:** chemical carcinogens, exposure, environmental exposure, occupational exposure, genotoxic, workers, workplace exposure, bladder cancer, systematic review

## Abstract

**Background**: There is substantial evidence supporting the role of genetic alterations in chemically induced carcinogenesis. We analyzed the existing literature to gather data on genetic alterations linked to human carcinogens and their possible connection to genotoxic outcomes. The review emphasizes carcinogenic substances and occupational exposures identified as “carcinogenic to humans”. In particular, we searched for studies describing genotoxic alterations linked to agents and occupational exposures for which the International Agency for Research on Cancer has found sufficient evidence of an association with bladder cancer. **Methods**: The review was carried out in compliance with the PRISMA standards. A comprehensive search of the PubMed database was conducted to identify studies published through March 2024. **Results**: We identified 60 studies that evaluated genetic alterations for 16 carcinogenic agents and occupations (such as aluminum production, 4-aminobiphenyl, auramine production, benzidine, chlornaphazine, cyclophosphamide, firefighters, magenta production, 2-naphthylamine, opium consumption, ortho-toluidine, painters, the rubber manufacturing industry, *Schistosoma haematobium* infection, X-radiation, gamma-radiation) in healthy humans. **Conclusions**: The genotoxic effects of chemical agents in healthy individuals have been well studied and characterized. Additionally, this review presents numerous studies concerning occupational exposure but not exclusively. Genotoxicity assessments have mainly been conducted on biological materials such as blood, peripheral blood lymphocytes, urine, and buccal epithelial cells. The most frequently examined genotoxic effects were DNA damage, chromosomal abnormalities, and micronuclei. Standardized data to clearly define a dose–response relationship for predicting delayed health effects are still lacking.

## 1. Introduction

As of 2022, bladder cancer ranked as the tenth most frequently diagnosed cancer globally [1]. The major risk factors encompass being male, advanced age, smoking, and contact with chemical carcinogens through occupational or environmental exposure [2]. Shifts in the demographic patterns of bladder cancer mirror both a growing awareness of the exposure risks and the ongoing transformation of industrial practices [3]. Occupational exposure to carcinogens is the second most common risk factor for bladder cancer in industrialized countries, accounting for approximately 5.7% of newly diagnosed cases [2]. Extensive research has explored the association between workplace exposure and cancer development, particularly highlighting links between bladder cancer and substances labeled as “carcinogenic to humans” (group 1) by the International Agency for Research on Cancer (IARC). Such carcinogens include 4-aminobiphenyl, benzidine, and 2-naphthylamine, as well as chemicals used in aluminum, auramine, and magenta production. The highest incidence ratios of bladder cancer have been reported among chimney sweeps, cooks, drivers, hairdressers, printers, seamen, stewards, and waiters [4]. Moreover, smoking is a very important risk factor that coexists with other factors. The mechanisms related to the development of bladder cancer in smokers are direct DNA damage, oxidative stress, DNA adduct formation, and epigenetic changes, and they affect the course of treatment.

The predominant form of bladder cancer in industrialized countries is urothelial carcinoma, accounting for more than 90% of diagnosed cases. This type of carcinoma often exhibits diverse histological variants. Around half of all urethral cancer cases originate as secondary tumors from urothelial carcinomas of the upper urinary tract or bladder. Environmental and occupational factors influence the development of specific types of bladder cancer. Urothelial carcinoma is strongly associated with smoking (carcinogens are excreted by the kidneys and come into contact with the urothelium) and with exposure to aromatic amines (which cause genotoxicity and lead to TP53 and FGFR3 mutations), as well as arsenic compounds in drinking water (which induce oxidative stress and impair DNA repair), ionizing radiation (which causes mutations and DNA damage), and cyclophosphamide. Squamous cell carcinoma is linked to *Schistosoma haematobium* infection, chronic urinary tract infections, urinary retention, and chronic irritation from catheters or bladder stones [5].

Cancer arises through a multifaceted, stepwise progression marked by the build-up of distinct tumor-related traits, many of which are heritable. The fundamental and sustained mechanisms driving tumor development and metastasis include persistent cell proliferation, the evasion of apoptosis, resistance to growth inhibition, an unlimited replication potential, chronic inflammation, altered metabolic pathways, genomic instability, the formation of new blood vessels, and enhanced invasiveness [6]. Notably, genotoxicity has been identified as a crucial feature among the ten key characteristics (KCs) of confirmed human carcinogens [7]. Any of these ten characteristics can interact with one another, providing stronger evidence for a cancer mechanism than each characteristic alone. In particular, one of the essential properties of an agent is its potential genotoxicity (KC2). Moreover, carcinogenic mechanisms are conceptualized based on these ten KCs, which correspond to 24 relevant toxicological endpoints (TEs) [7,8]. These TEs are associated with cancer induction, including cellular and molecular changes that are linked to different stages of carcinogenesis [8,9].

In the 1970s, the IARC published its first monograph, Evaluation of Carcinogenic Risk of Chemicals to Man Volume 1 [10]. Many agents classified as group 1 human carcinogens were reviewed over 50 years ago, when mechanistic studies were not included in the carcinogenicity assessment. Later studies revealed that many of these previously identified cancer hazards may lead to cancer in additional organs, depending on the nature of the exposure [11]. Since then, a database of the mechanistic properties of human carcinogens has been established by compiling mechanistic information on agents identified as group 1 in the IARC Monographs on human carcinogenic risk assessments. Section 4 of the IARC Monographs, titled Mechanistic and Other Data, provides a concise summary of relevant data on the mechanisms of carcinogenesis for the agent under review, based on studies in humans and experimental animals and in vitro. So far, the IARC has identified 128 agents as belonging to group 1. Among these, the largest group consists of “chemical agents and related occupations,” comprising 45 agents (35%). Notably, nearly one in four “chemical agents and related occupations” with sufficient evidence for cancer in humans also causes bladder cancer. Among the 19 agents strongly associated with bladder cancer in humans, a significant proportion (10 agents, or 53%) fell under the category of “chemical agents and related occupations”.

The IARC classifies the urinary tract into the following cancer sites: the kidney, renal pelvis, ureter, and urinary bladder. Among the urinary bladder carcinogenic agents with “sufficient evidence” in humans, 19 were identified (Table 1). These correspond to a group 1 IARC classification for the following risk factors in bladder cancer, including tobacco smoking, various occupational agents (4-aminobiphenyl, 2-naphthylamine, ortho-toluidine), various occupations (aluminum production, rubber manufacturing, dye manufacturing (benzidine), the dye industry (auramine and magenta production), firefighting and painting, medications or drugs (chlornaphazine, cyclophosphamide, and opium consumption), environmental factors (arsenic and inorganic arsenic compounds, X- and gamma-radiation), and disease (*S. haematobium* infection) [12]. Eight of the listed causes of bladder cancer are precipitating factors in other cancer sites with sufficient or limited evidence in humans.

This review concentrated on human bladder cancer carcinogens, as classified by cancer site classifications, with sufficient evidence in humans from IARC Monographs Volumes 1–135 [13]. However, because of the extensive number of studies within the scope of our research, three risk factors—arsenic and inorganic arsenic compounds (environmental factors) and tobacco smoking—were excluded from this systematic review.

Through the integration of existing scientific findings, we developed an in-depth summary highlighting the patterns and relevance of genotoxic effects, drawing on data from studies examining various exposures associated with bladder cancer. Specific examples are provided for the genotoxic alterations caused by 16 individual agents (e.g., aluminum production; 4-aminobiphenyl, 2-naphthylamine, ortho-toluidine, benzidine, auramine, and magenta production; firefighting; painting; rubber manufacturing; chlornaphazine; cyclophosphamide; opium consumption; X-radiation; gamma-radiation; *S. haematobium* infection).

## 2. Materials and Methods: Search Strategy and Selection Criteria

**Figure 1:**
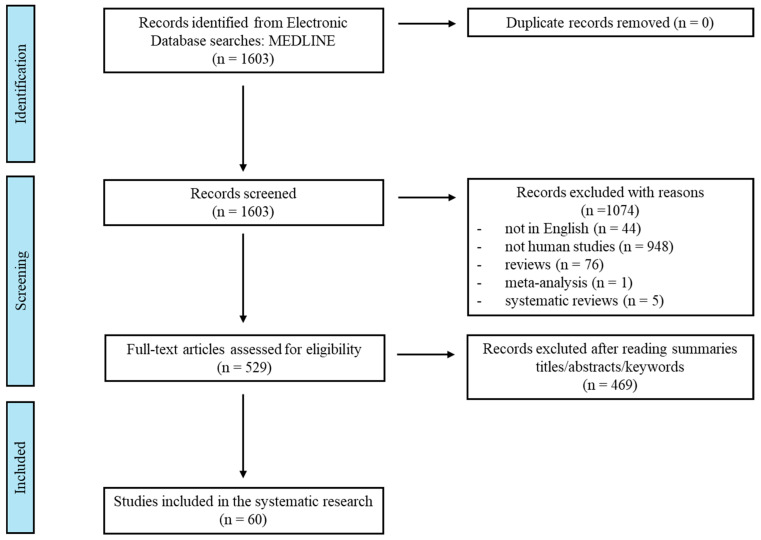
Flowchart outlining the systematic review process.

We carried out a comprehensive search in the PubMed database for publications up to March 2024, employing both subject headings and keyword searches. Studies were included based on the following criteria: population, healthy general and occupational population, exposure to carcinogenic agents, or occupational exposure with sufficient evidence for bladder cancer by the IARC. The final fields search syntax was as follows: ((Genotoxic) AND ((Aluminum) OR (4-Aminobiphenyl) OR (Auramine) OR (Benzidine) OR (Chlornaphazine) OR (Cyclophosphamide) OR (Firefighter) OR (Magenta) OR (2-Naphthylamine) OR (Opium) OR (Paint) OR (Painter) OR (Rubber) OR (*Schistosoma haematobium*) OR (Ortho-Toluidine) OR (X and Gamma-radiation)). The characteristics of the included studies are shown in Figure 1. The search was limited to English-language articles published in peer-reviewed journals. Studies were excluded if they were non-English, non-human, contained abstracts only, contained comments, or were conference papers. Two independent reviewers (E.K. and K.T.) screened the titles and abstracts, followed by full-text assessments. Any disagreements about a study’s inclusion were resolved through a discussion with a third author (E.J.). Additionally, the reference lists of all selected articles concerning the link between human bladder cancer carcinogens and genetic alterations were manually examined to identify further relevant studies. This systematic review adhered to the Preferred Reporting Items for Systematic Reviews and Meta-Analyses (PRISMA) guidelines [14].

## 3. Results

**Figure 2:**
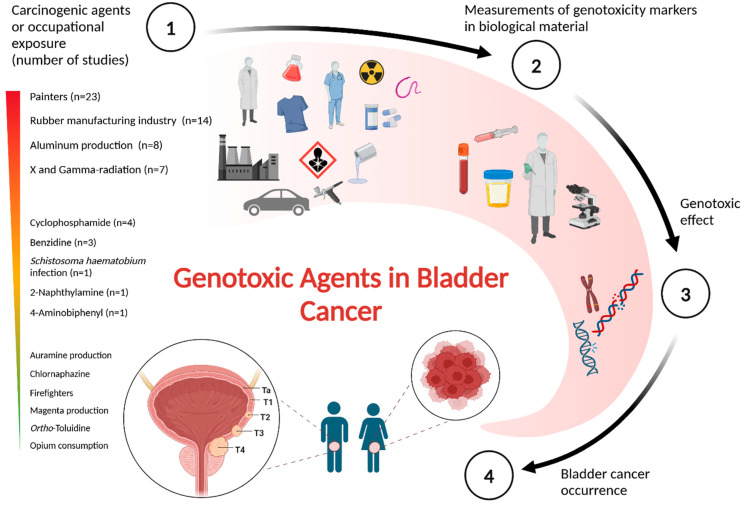
Genotoxic agents in bladder cancer. A visual overview presenting the key assumptions and findings from the literature review regarding genotoxicity caused by carcinogenic agents or occupational exposures with strong evidence for bladder cancer. The figure was generated using BioRender.com.

Following the removal of duplicates, a total of 1603 studies were identified, with 529 available as full-text articles. A review of the literature on genotoxic exposure to carcinogenic agents and occupations showed that occupational exposure was the most frequently researched factor (n = 60) (Figure 2). Much research has been conducted on painters (n = 23), the rubber manufacturing industry (n = 14), and aluminum production (n = 8). Several studies have been conducted on X- and gamma-radiation (n = 7), cyclophosphamide (n = 4), and benzidine (n = 3). However, few studies have focused on human genetics and *S. haematobium* infection (n = 1) or 4-aminobiphenyl and 2-naphthylamine (n = 1). No research has been directly related to auramine production, chlornaphazine, firefighters, magenta production, opium consumption, and ortho-toluidine.

Below is a description of each of the 16 human carcinogens that fulfilled the inclusion criteria, followed by an analysis of the genotoxicity findings from the reviewed studies (Table 2). Among these, only 11 carcinogenic agents or occupations had 60 studies reporting on KC2.

### 3.1. Occupational Exposure in Aluminum Manufacturing

Aluminum ranks among the most extensively produced metals worldwide, with annual production exceeding 69 million metric tons by December 2022, up from 67.5 million metric tons the year before. This positions aluminum as the second most produced metal, following steel [75]. Although aluminum is the third most abundant element in the Earth’s crust, following oxygen and silicon, it does not naturally occur in its metallic form [76]. The aluminum manufacturing sector, linked to exposure to polycyclic aromatic hydrocarbons (PAHs), is classified as a group 1 carcinogen. Among the leading aluminum producers, one utilized prebake technology, whereas another employed a modified Søderberg process. The common job functions in aluminum smelting plants involve operating cranes and vehicles, conducting tapping and anode replacements, monitoring pots, and maintaining pot linings [77].

The genotoxic impact of exposure to coal tar pitch volatiles has been studied among workers at an aluminum reduction facility. In this study, a series of tests were conducted on three distinct body fluids—urine, blood, and semen. In the exposed group, mechanics exhibited a higher prevalence of mutagenic urine than in the other worker categories. The frequency of chromosomal aberrations was largely comparable between exposed and unexposed workers. The semen analysis results did not reveal any disparities between the exposed and non-exposed workers [15].

The results of a comparative study performed in two aluminum plants highlight the need for ongoing biomonitoring studies to identify potential changes in biological effects caused by evolving exposures. The level of aromatic DNA adducts has been determined by a 32P-postlabeling assay and enzyme-linked immunosorbent assay in peripheral blood lymphocytes collected on two occasions, one year apart, and could provide improved insight into the strengths and limitations of the two methods used in these studies [16]. The 32P-postlabelling method has some limitations, as it does not give structural information and has poor reproducibility. Further longitudinal human biomonitoring studies performed in two Hungarian primary aluminum production plants that operated Söderberg cells by the same group of scientists indicated that carcinogen–DNA adducts can serve as effective biomarkers for assessing occupational exposure to genotoxic PAHs [17]. Additionally, a Hungarian study evaluated the sensitivity, specificity, and correlations between various biomarkers used to monitor occupational exposure to complex mixtures of genotoxic agents. These findings reaffirm earlier observations, indicating a lack of correlation between DNA adducts detectable by 32P-postlabeling and those measured via the PAH-DNA immunoassay within the same DNA sample. Additionally, these studies emphasized a weak correlation between DNA adduct levels and urinary 1-hydroxypyrene (1-OH-PY) concentrations within the same individuals [18].

Employees working in potrooms at aluminum reduction facilities are at increased risk of developing bladder and lung cancers as a result of exposure to PAHs. This study examined airborne PAH exposure in pools with genotoxic or mutagenic effects and evaluated how different host genotypes of metabolizing enzymes might influence the association between PAH exposure and genotoxic or mutagenic responses. Although potroom workers involved in aluminum reduction were significantly exposed to PAHs, there was no observed elevation in aromatic DNA adducts in lymphocytes, despite the correlation between most airborne PAH congeners and the excretion of 1-hydroxypyrene in urine [19]. Samples of urine were gathered from male workers in potrooms and from unexposed blue collar workers for the purpose of measuring their 8-hydroxydeoxyguanosine concentrations. Additionally, various analyses were conducted on peripheral mononuclear cells, including the evaluation of DNA single-strand breaks; micronuclei in CD4+ and CD8+ lymphocytes; the hypoxanthine guanine phosphoribosyl transferase mutation frequency; microsomal epoxide hydrolase; the genotype for cytochrome P-4501A1; and glutathione transferases M1, T1, and P1. The findings from this study suggest that the selected biomarkers for mutagenic or genotoxic effects may not be suitable for monitoring potroom workers exposed to present-day airborne PAH concentrations [20].

The phase I and phase II xenobiotic-metabolizing enzyme families are essential in both activating and detoxifying various environmental carcinogens. Certain genetic polymorphisms in these enzymes have been found to influence an individual’s cancer susceptibility. Understanding the impact of these metabolic gene interactions may be improved by grouping individuals according to various factors that could affect the observed biomarker outcomes. Additionally, using biomarker methods specific to chemical structures may provide further clarity. Scientists have investigated the impact of various interactions between *CYP1A1* Ile462Val, *CYP1A1* MspI, *CYP1B1* Leu432Val, *CYP2C9* Arg144Cys, *CYP2C9* Ile359Leu, *NQO1* Pro189Ser, *GSTM1* gene deletion, and *GSTP1* Ile105Val genotypes on the levels of carcinogen–DNA adducts detected via 32P-postlabeling and PAH-DNA immunoassays. This investigation was conducted on peripheral blood lymphocytes from workers with occupational exposure to PAHs in aluminum plants, as well as on bronchial tissue from lung cancer patients who were smokers. A statistically significant positive linear correlation was observed between the levels of aromatic DNA adducts in white blood cells and urinary (1-OHPY) levels in potroom operators with the *GSTM1* null genotype. These findings suggest that interactions between *GSTM1* and *GSTP1* alleles modulate the levels of urinary 1-OHPY and aromatic DNA adducts in white blood cells among PAH-exposed workers [21].

The elevated levels of metals found in body fluids, along with the presence of DNA fragmentation and chromosomal abnormalities in lymphocytes, underscore the necessity for the implementation of safety initiatives tailored specifically for welders. Iarmarcovai et al. investigated the occupational hazards faced by welders by examining the metal levels in biological fluids (including blood and urine, to measure levels of aluminum, cadmium, chromium, cobalt, lead, manganese, nickel, and zinc), assessing DNA damage using various genotoxic endpoints and analyzing polymorphisms in DNA repair genes. They discovered statistically significant variances between welders who worked without collective protective measures and those with smoke extraction systems, particularly regarding their blood cobalt concentrations and urinary levels of aluminum, chromium, lead, and nickel. The alkaline comet assay demonstrated a notable increase in OTMchi2 distribution among welders by the end of the week compared to the start, indicating a significant increase in DNA strand breaks in most welders. In addition, the cytokinesis-block micronucleus assay revealed that the welders without collective protective measures exhibited a greater incidence rate of chromosomal damage than the control subjects. Furthermore, the XRCC1 variant allele encoding the Gln amino acid at position 399 is linked to a higher frequency rate of DNA breaks, as evidenced by the comet assay [22].

### 3.2. Aromatic Amines: 4-Aminobiphenyl, 2-Naphthylamine, and Ortho-Toluidine

The toxicity characteristics of aromatic amines (such as 2-naphthylamine and 4-aminobiphenyl) are primarily associated with metabolic activation of the amino group and production of reactive intermediates, which form DNA adducts and may lead to carcinogenic effects. The levels of 8-hydroxy-2′-deoxyguanosine (8OHdG) in urine serve as a significant biomarker of DNA damage. These findings suggest that regulated aromatic amines can induce DNA damage, increasing the levels of 8OHdG [23].

Exposure to 4-aminobiphenyl primarily occurs in occupational settings, where it is used as an intermediate in dye production and an antioxidant in rubber. Since the industrial production of this amine ceased in 1955, the current exposure rate has arisen from the contamination and metabolic release of benzidine. This amine is also a byproduct of tobacco combustion and has been detected in kitchen oil fumes. Bladder cancer is specifically associated with exposure to 4-aminobiphenyl. The incidence of this cancer has been reported in chemical plant workers and experimental animal models. Like other amines, this compound is metabolically activated through various pathways to produce reactive intermediates that interact with DNA, leading to mutations. Scientists have detected 4-aminobiphenyl-DNA adducts in the bladder tissues of exposed individuals.

2-Naphthylamine is also an aromatic amine, which is a polar organic chemical substance with a wide range of environments. Various potential sources of exposure (such as tobacco smoke, diesel exhaust, and dermal absorption from textile products with azo dyes) are known, resulting in potential harm to human health. For example, the nitrogen content of tobacco leaves, which significantly contributes to the formation of aromatic amines, and tobacco smoke inhaled by smokers and non-smokers have been identified as important sources of exposure [23].

Ortho-toluidine, used as an intermediate in the dye industry and also applied in sectors such as rubber processing, pharmaceutical manufacturing, and herbicide production, has been produced for more than 150 years. Exposure predominantly occurs in occupational environments, with non-occupational exposure mostly linked to smoking and the use of hair dyes. There is a well-established link between exposure to ortho-toluidine and an increased risk of bladder cancer in humans. Since the 1990s, partly based on in vivo research, it has been recognized that ortho-toluidine is metabolized into several compounds that are also genotoxic. The specific metabolic pathway examined in this study appears particularly important. Ortho-toluidine has demonstrated carcinogenic effects in mice and rats, with suspected similar impacts in humans. The research involving bacteria, fungi, and mammals has shown that ortho-toluidine can act as a mutagen. It also causes DNA damage, such as single-strand breaks and unscheduled DNA synthesis, leading to cell transformation [78]. In one study, Suzuki et al. treated DNA from rat bladder epithelial cells with aromatic amines, including acetoacet-ortho-toluidine and ortho-toluidine, and conducted an adductome analysis. Their findings suggested that oxidative stress, indicated by oxidative DNA adducts such as 8-hydroxy-2′-deoxyguanosine (8-OHdG), may play a role in the development of urinary cancer caused by ortho-toluidine [79].

### 3.3. Auramine Production

Auramines belong to the family of aromatic amines and share common carcinogenic mechanisms; however, their metabolism has not been thoroughly investigated. In experimental animals, auramine induced hepatocellular carcinoma and lymphoma. Individuals exposed to auramines were primarily involved in the production of this dye. It has been identified that the production of auramine causes bladder cancer in humans. In addition, auramine production is associated with high exposure to aromatic amines. Auramine and its salts are mainly used in the production of dyes, primarily for paper staining. However, the use of this substance has been banned in many countries. According to the IARC, in vivo experimental studies in animals have shown that auramine causes DNA strand breaks. In Saccharomyces cerevisiae, auramine induces genotoxic effects by generating free radicals. A genotoxic effect was identified when auramine-induced intrachromosomal recombination in *S. cerevisiae* decreased in the presence of the free radical scavenger N-acetylcysteine. Additionally, in vitro experiments have also revealed DNA strand breaks, abnormal DNA synthesis, micronuclei development, and the induction of deletions and aneuploidy in *S. cerevisiae*. Mutagenic activity has also been demonstrated in several strains of S. typhimurium using metabolic activation systems. There is currently a lack of mechanistic data regarding the carcinogenicity of auramine in humans [80,81]. Auramine yielded a positive Ames test result. Studies on the mechanism of genotoxicity conducted using human lymphoblastoid TK6 cells lacking XRCC1 and XPA (XRCC1-/-/XPA-/-), which are key factors in base excision repair (BER) and nucleotide excision repair (NER), respectively, suggest that auramine is a genotoxic agent that preferentially induces DNA damage repair in mammals via BER, NER, or both [82].

### 3.4. Benzidine Exposure

Benzidine is primarily used as a base for various dyes applied in textiles, as well as for visually detecting blood cells under laboratory conditions. Workers exposed to benzidine experience increases in both the occurrence and fatality rates of bladder cancer [83]. The risk of developing bladder cancer and succumbing to it remains heightened even more than two decades after an individual’s last exposure to benzidine, particularly among those employed for over five years. This aromatic amine can be metabolized into DNA-reactive intermediates, potentially leading to chromosomal aberrations, DNA strand breaks, micronuclei formation, DNA adducts, and mutations in oncogenes. Bladder cancer is a primary cancer associated with occupational exposure to benzidine. It is a multifocal carcinogen that primarily induces liver cancer [84]. Uziel et al. in a review article provided an overview of DNA adduct formation and method detection and the route of absorption, metabolism, and chemistry of hazardous chemicals, such as benzidine and benzidine dyes (Direct Blue 6, Direct Black 38, and Direct Brown 95). They discussed possible complicating factors consisting of combined exposures within the workplace, as well as the creation of shared DNA adducts [85]. Research carried out on 32 workers exposed to benzidine in India revealed that the urine pH significantly affects the presence of free urinary aromatic amine compounds and levels of DNA adducts in urothelial cells [25].

A cross-sectional investigation involving 33 workers exposed to benzidine and 15 unexposed controls in India assessed the presence of benzidine-related DNA adducts in exfoliated urothelial cells, excretion patterns of benzidine metabolites, and the influence of NAT2 activity on these outcomes. This study suggests that benzidine forms DNA adducts in exfoliated urothelial cells of exposed individuals, with the predominant adduct being N-acetylated, which supports the notion that monofunctional acetylation is an activating step rather than a detoxification process for benzidine. The NAT2 activity did not affect the levels of any measured DNA adducts, indicating that inter-individual variation in NAT2 function is unlikely to be relevant for benzidine-associated bladder carcinogenesis [24].

A cross-sectional study conducted on 30 workers exposed to benzidine (low and high exposure levels) and 13 unexposed control groups validated the effectiveness of using biomarkers, such as urinary mutagenicity, to detect low-level exposures and uncover additional genotoxic exposures within the control group, and demonstrated a strong correlation with urinary metabolites and DNA adducts in the target tissue (urinary bladder epithelium) in humans [26].

### 3.5. Chlornaphazine Treatment

In addition, chlornaphazine has been associated with an increased risk of bladder cancer [86]. This 2-naphthylamine derivative has been previously used to treat polycythemia and Hodgkin’s disease. A higher incidence rate of bladder cancer was first observed in patients with polycythemia who were treated with chlornaphazine than in those who were not; later, an increased risk was also noted in patients treated for Hodgkin’s disease with this drug [87,88]. No studies have directly assessed the influence of chlornaphazine exposure on the potential genotoxic mechanisms in healthy human subjects.

### 3.6. Cyclophosphamide Exposure

Cyclophosphamide is a widely recognized anticancer medication with established genotoxic effects across various testing platforms. The genotoxic effects on non-tumor cells are particularly noteworthy because they can potentially trigger secondary tumors in cancer patients. Cytostatic medications, which are undeniably beneficial in cancer therapy, pose significant risks to healthcare workers in occupational environments owing to their high cytotoxicity. Cyclophosphamide, an antineoplastic medication employed in cancer therapy, poses inherent risks due to its genotoxic, teratogenic, and carcinogenic characteristics. Its use is acknowledged as an occupational danger for healthcare professionals who may encounter it. Substantial contamination was identified on different surfaces within the pharmacy and oncology departments, indicating possible sources of exposure. Personnel in oncology units experience exposure more frequently than those in pharmacy units [89]. In a workplace setting, concurrent exposure to cytostatic drugs may increase the risk compared with exposure to a single substance alone. These findings indicate that a combination of cytostatic drugs has the potential to cause cellular and genetic damage, even at minimal concentrations. Gajski et al. suggested that not only can such a combination pose a threat to the integrity of cells and genomes but also that toxicity data from individual compounds alone are insufficient for predicting toxicity in a complex occupational setting [90]. Heightened genetic damage was apparent among nurses at the population level and was attributed to occupational exposure to antineoplastic drugs [27].

These findings validated the genotoxic effects of antineoplastic medications on peripheral blood lymphocytes. Additionally, in exfoliated buccal cells, the results indicate more consistent genetic harm occurring during the administration of antineoplastic drugs than during their preparation [28]. Ursini et al. showed that the comet assay, known for its sensitivity in detecting the early effects of recent exposure to genotoxic substances, revealed only minor DNA damage in exfoliated buccal cells of hospital day nurses, specifically in the group responsible for administering the highest quantity of drugs during the administration process. They proposed that utilizing the comet assay on exfoliated buccal cells could serve as a valuable method to assess the early and potentially reversible genotoxic effects resulting from exposure to combinations of antineoplastic drugs, thereby aiding in enhancing hospital safety protocols [29]. A study assessing genotoxicity in oncology nurses from a South Indian hospital found increased genetic damage linked to exposure to antineoplastic drugs at work [30].

### 3.7. Occupational Exposure Among Firefighters

Firefighting involves numerous factors that can affect both the level and type of occupational exposure experienced. Firefighters face a complex mix of chemical, physical, biological, and psychosocial risks stemming from their duties, which include active firefighting, training drills, fire management, and the protection of life and property during emergencies. They respond to a wide range of incidents such as fires of various origins, vehicle crashes, medical emergencies, hazardous material releases, and structural collapses. During these events, firefighters may be exposed to multiple harmful substances found in smoke, such as formaldehyde, acetaldehyde, benzene, toluene, sulfur dioxide, and ethylbenzene [2]. Studies indicate that firefighters can be exposed to up to 27 group 1 carcinogens, contributing to increased rates of various cancers [91].

### 3.8. Magenta Production

Studies indicate that firefighters can be exposed to up to 27 group 1 carcinogens, contributing to increased rates of various cancers and possessing common carcinogenic mechanisms with other members. A direct examination of magenta dye metabolism has not yet been conducted, and there is no readily available information regarding the specific carcinogenic mechanisms associated with magenta. Nevertheless, workers involved in manufacturing these dyes, such as magenta I, II III, and 0 (Basic Red 09), are the major exposed group in which we observe the occurrence of bladder cancer [80]. The exposure of workers to various aromatic amines in the workplace makes it difficult to assess the carcinogenicity of magenta itself. However, in two small cohorts of workers involved in magenta production, a significant excess risk of bladder cancer was observed [92].

### 3.9. Opium Use

In 2020, the IARC Working Group evaluated the carcinogenic potential of opium and classified its consumption as “carcinogenic to humans.” They highlighted strong evidence linking opium use to cancers, especially bladder, laryngeal, and lung cancers. Research conducted in Iran found that habitual opium consumption was associated with roughly a four-fold higher risk of developing bladder cancer (OR 3.5, 95% CI: 2.8–4.3). Furthermore, a combined effect was observed for users of both opium and tobacco, with an OR of 7.7 (95% CI: 6.0, 9.7) [93].

### 3.10. Occupational Exposure as a Painter

Paint products contain thousands of compounds with various functions. These substances or emission sources include antifouling agents; specialized waterproof coatings for tanks, ships, and pipes; pigments; solvents such as white spirits and naphthas; paint removers; binders used in special and water-based paints; sandblasting materials; fillers; and compounds used in taping and spackling, such as talc. In recent years, many hazardous chemicals, including benzene, chromium, lead, and phthalates, have been reduced or removed from paint formulations. The increased cancer risk may stem from the complexity, changing composition, quantities, and possible interactions of these chemical mixtures.

Genotoxicity has been identified as one of the mechanisms that contribute to the documented increase in cancer risk. The presence of various genetic and cytogenetic abnormalities among painters and paint industry workers provides convincing proof of genotoxicity [94]. The genotoxic effects of occupational exposure among painters are ascribed to the genotoxic properties of specific components found in paints, such as benzene, toluene, styrene, and PAHs [31]. The research indicates an association primarily between cancer sites, such as lung and bladder cancers, and occupational exposure to painters. Associations between childhood leukemia and maternal exposure during painting have also been reported. Prolonged exposure to paint in the workplace could result in a minor increase in the risk of genetic damage among individuals employed in the paint industry. Occupational painters such as outdoor and automobile painters have been reported to exhibit chromosomal abnormalities, elevated micronuclei levels, and increased sister chromatid exchanges [32,33]. Madhavi et al. reported a notable rise in the incidence of chromosomal aberrations among industrial painters compared to the control group [34]. Evaluating DNA damage in various auto body shop employees through micronucleus levels in exfoliated buccal cells revealed that technicians and painters are susceptible to genotoxic harm, while office workers appear to be less affected [35]. Investigations have highlighted the application of the comet assay in an occupational exposure study of genotoxic agents. Genotoxicity assessed using the comet assay in peripheral blood leukocytes and buccal epithelial cells revealed that both the damage index and damage frequency were markedly elevated in the exposed group compared to the control group [36]. The results showed an increase in DNA breaks in automotive paint technicians [37] and the construction painters group [38]. Pereira da Silva et al. suggested that car painters comprise a high-risk group because paints can induce genotoxic and mutagenic effects in the peripheral blood and oral mucosa cells, respectively [39]. Londoño-Velasco et al. observed that exposure to organic solvents and paints led to elevated levels of oxidative damage in the DNA of lymphocytes among auto body painters, including the generation of 8-oxodG and other formamidopyrimidine products known for their high mutagenic potential [40]. Cetintepe et al. evaluated the genotoxic effects, oxidative stress levels, and immune responses in workers involved in automotive painting. They concluded that workplace exposure to chemicals in the automotive sector could lead to DNA damage in employees as a result of oxidative stress [41]. Studies among groups of painters have compared oxidative stress and DNA damage biomarkers with specific volatile organic compounds [42]. Moreover, the study conducted by KanuPriya et al. presented compelling evidence supporting the identification of a direct correlation between glutathione-S-transferase polymorphisms and genetic injury detected in employees of the paint sector [43]. Hoyos-Giraldo et al. reported elevated DNA methylation levels in the promoter regions of the GSTP1 and p16INK4a genes in exfoliated urothelial cells from car painters exposed to occupational hazards, compared to the reference values. Furthermore, these alterations in genes are associated with an increase in the occurrence of micronuclei, suggesting the presence of genotoxic effects [44].

### 3.11. Rubber Manufacturing Industry

Occupational exposure in the rubber industry not only causes tumors such as bladder, lung, and stomach cancers but also leukemia and lymphoma. Employees employed in the rubber industry are potentially exposed to various chemical substances with different compositions, depending on the production process, such as hydrocarbons, N-nitrosamines, polycyclic aromatic, phthalates, and solvents [95]. The main sources of exposure are dust and vapor generated during various production processes. The primary route of exposure for workers is through the respiratory system but also often through the skin, such as for cyclohexane-soluble compounds. Due to the variable and complex nature of the mixture, as well as possible interactions between exposures, various carcinogenic mechanisms play a role in increasing the risk of cancer. Carcinogenic mechanisms reflect the mechanisms of specific chemical exposures. The main mechanisms include genotoxicity, which has been confirmed by various genetic and cytogenetic effects. Studies conducted among rubber industry workers have shown chromosomal aberrations, sister chromatid exchanges, micronuclei formation, early chromosome condensation, DNA breakage, DNA damage, the formation of DNA adducts, HPRT gene mutations, and mutagenic activity in urine samples [80,96].

The toxicology of the chemicals used in the rubber industry has been studied for several years. Employees at rubber tire plants, who are exposed to various pollutants such as acetonaphthene, alkenes, benzo[a]pyrene, benzo-fluoranthene, 1,3-butadiene, and naphthalene, have been observed on a regular basis for many years. This monitoring included assessments of chromosomal abnormalities in lymphocytes, the mutagenicity of urine using the Ames test, and various blood and urine parameters. Scientists have used biological monitoring methods to identify categories of job positions to investigate the existing risk of exposure to potential genotoxic chemical substances in the work environment. High mutagenic activity has been found in urine samples of workers from various departments of the rubber industry, as well as in vulcanizers. Meanwhile, sister chromatid exchanges and an increase in chromosomal aberrations have been observed in peripheral blood samples [54]. Analyses of lymphocytes from clinically healthy workers employed in the rubber industry indicated an increase in the frequency of chromosomal aberrations and sister chromatid exchanges, as well as a decrease in proliferation indices. Occupational exposure, age, and smoking history were correlated with cytogenetic parameters. The results suggested an exacerbation of genotoxic effects due to combined exposure to chemical substances and cigarette smoking [55]. A further cytogenetic investigation by this group of scientists did not confirm that smoking cigarettes enhances the genetic effects of chemicals in the rubber industry [56]. The results of biological monitoring for genotoxic exposure in the rubber industry revealed statistically higher mean values for indicators of induction of the microsomal enzymatic system (phase I) of 17-hydroxycorticosteroids and micronuclei, and lower values of 6-beta-hydroxycortisol compared to the control group, taking smoking into account. These results indicate that working in the rubber processing industry may lead to genetic alterations and influence the function of specific enzymes [57]. The research conducted by Somorovská et al. showed significantly elevated levels of DNA strand breaks compared to office workers, as well as in the laboratory control group. In the exposed group, micronuclei were observed at notably higher rates than in the control group, although all frequencies fell within the expected range. Notable correlations were observed between individual measurements of strand breaks, micronuclei, chromatid and chromosome breaks, and specific immunological parameters [59]. Studies assessing the sensitivity, specificity, and correlation between multiple biomarkers used to monitor occupational exposure to various genotoxic factors in the work environment did not show a strong correlation between individual biomarkers, such as DNA adducts detectable by 32P-postlabeling, and those measured using the PAH-DNA immunological test, DNA adduct levels, and 1-hydroxypyrene levels in urine [18]. 1,3-Butadiene, used in the production of synthetic rubber, is a mutagenic and carcinogenic substance. Exposure to 1,3-butadiene has genotoxic effects, resulting in an increased frequency of lymphocytes harboring mutations in the hprt reporter gene. Additionally, the frequency of mutated hprt cells is correlated with the concentration of 1,3-butadiene in the air and the concentration of 1,3-butadiene metabolites in urine. The impact of exposure on the frequency of mutated hprt lymphocytes has been repeatedly observed in studies conducted in Texas [60]. Further studies investigating the correlation between exposure to 1,3-butadiene and biomarkers of internal exposure and genotoxicity indicate that elevated workplace exposure to 1,3-butadiene could be linked to harmful biological outcomes [61]. The research on the relationship between inhalation and dermal exposure and the presence of mutagenic activity in the urine of rubber industry workers indicates that dermal contact may play a greater role than inhalation in contributing to urinary genotoxic compound levels [63]. Studies indicate that employees working in the compounding, mixing, and curing departments face the highest risk of genotoxicity among rubber manufacturing workers. Elevated levels of urinary 1-hydroxypyrene, mutagenicity, and DNA adducts in the urothelial cells were observed in these workers. The occurrence of particular bladder-cancer-causing agents in the rubber production sector is suggested by the lack of correlation between urothelial cells and peripheral blood mononuclear cell DNA adducts [64].

Styrene is widely used in the production of synthetic plastics, polyesters, resins, and rubbers. A robust and statistically significant association was identified between environmental styrene levels and its metabolites in urine (levels of mandelic acid and phenylglyoxylic acid in urine). The results from the genetic damage tests using the micronucleus test and comet assay showed slight and insignificant increases associated with exposure. A higher rate of sister chromatid exchange was noted in the exposed workers than in the control group. Furthermore, significant correlations were obtained between the sister chromatid exchange rate and the exposure parameters [65]. Premature centromere division—referring to the early separation of centromeres during the prometaphase or metaphase of mitosis—may represent a form of chromosomal instability observed in human chromosomal breakage syndromes. Such instability is frequently detected in the peripheral blood lymphocytes of individuals occupationally exposed to clastogenic agents and is regarded as a contributing factor in cancer development. Studies on the induction of premature centromere division in peripheral blood lymphocytes of workers exposed to various genotoxic agents—such as acrylonitrile, dimethylformamide, or benzene; a combination of exposures in the rubber industry; and an organic solvent mixture including CCl4, tar, hot oil mist, and polychlorinated biphenyls—have shown that induction is not random but related to occupational exposure [58]. Bladder cancer is considered a historic disease among rubber industry workers who are occupationally exposed to aromatic amines such as 2-naphthylamine. Despite the decline and limitations of occupational exposure to these compounds, a directly proportional decrease in the risk of bladder cancer has not been observed. Studies have reported elevated average levels of 2-napthol in the urine of workers. However, the levels of 2-napthol did not accurately forecast the amount of carcinogen DNA adducts in exfoliated urothelial cells [66].

### 3.12. Chronic Infection with Schistosoma haematobium

*Schistosoma haematobium*, a parasitic organism commonly found in tropical areas of Africa and the Middle Eastis, is linked to the formation of squamous cell carcinoma in the bladder. Although carcinogenesis is probably affected by various factors, it has been proposed that these parasites produce or release oxysterols and metabolites possessing estrogen-like effects, which may act as initiators of cancer development related to their infections [97]. The life cycle of *S. haematobium* includes reproduction inside the human body, with eggs being released through urine. These eggs then enter freshwater snails and eventually infect humans again when they come into contact with contaminated water. Upon reaching maturity, the mature parasite adheres to the veins located in the vicinity of the bladder, leading to the release of eggs through the bladder wall. Adult flukes further enhance cell proliferation and migration while reducing apoptosis. The development of carcinogenesis requires chronic infection. Moreover, simultaneous infections with bacteria and flukes have been found to increase the risk of developing bladder cancer.

Compounds found in *S. haematobium* extracts, known as catechol estrogens, exhibit estrogen-like properties. These substances have been observed to reduce the activity of estrogen receptors alpha and beta in estrogen-sensitive cells. Catechol–estrogens derived from schistosomes cause genotoxic effects, leading to the formation of estrogen–DNA adducts. These substances, along with the resulting catechol–estrogen–DNA adducts, can be detected in the blood of *S. haematobium*. Santos et al. indicated that catechol–estrogen–DNA adducts were significantly associated with schistosomiasis [67]. A pathway potentially mediated by estrogen–DNA adducts may contribute to the development of squamous cell carcinoma in the bladder, linked to infection with the blood fluke *S. haematobium*. Substances extracted from various developmental stages of *S. haematobium*, such as eggs, trigger cell cultures to exhibit tumor-like characteristics. Furthermore, reactive metabolites derived from estrogen are present in this parasite, as well as in the blood of infected individuals. An examination of urine samples from 40 Angolan individuals diagnosed with urogenital schistosomiasis detected several estrogen-like compounds, with seven identified exclusively in individuals with urogenital schistosomiasis, not previously documented in urinary metabolite databases from healthy individuals. The metabolites associated with schistosome infection include catechol estrogen quinones and their DNA adducts. Metabolites originating directly from 8-oxodG have been discovered in the urine of individuals with urogenital schistosomiasis [98].

### 3.13. X- and Gamma-Radiation

Everyone is exposed to ionizing radiation from natural background sources such as soil, building materials, cosmic rays, and radon. However, the average radiation exposure is increasing, primarily due to the growing use of medical imaging, especially the widespread use of computed tomography (CT). In developed countries, the rise in angiography and interventional radiology procedures has also contributed significantly to this increase. As a result, it is believed that medical procedures account for as much as 50% of the present-day exposure to external ionizing radiation. Investigations involving populations subjected to high levels of radiation from atomic bomb detonations have significantly enhanced our knowledge of cancer development and the mechanisms of radiation-induced carcinogenesis. Scientists have identified two primary theoretical frameworks to explain how ionizing radiation leads to cancer—the mutation theory, which involves alterations in DNA that lead to coding errors, and the theory of non-genetic effects, which focuses on epigenetic mechanisms. Mutation theory, being purely genetic in nature, posits that ionizing radiation causes DNA damage. When repair mechanisms fail to correct all DNA lesions, mutations can occur during cell division. The development of cancer is, thus, driven by the gradual buildup of mutations through the clonal growth of altered cells.

Ionizing radiation deposits energy into cellular macromolecules, resulting in various forms of DNA damage. This damage occurs in two ways—directly ionizing atoms in the DNA itself or indirectly causing ionization by interacting with free radicals, such as hydroxyl radicals, which are produced when radiation interacts with water molecules. Substantial evidence shows that ionizing radiation can cause a wide range of damages (including base pair damage, single-strand breaks, DNA–protein cross-links, sister chromatid exchanges, double-strand breaks, and their combinations) and DNA mutations, leading to large-scale gene deletions, severe chromosome damage, and genetic variability. The observed events include chromosomal aberrations, mutations in gene sequences, minisatellites, and apoptosis [80].

Visweswaran et al. assessed DNA damage and gene expression changes in the peripheral blood samples of patients undergoing neurointerventional radiological procedures and correlated these changes with the entrance surface dose. Their findings indicated that most patients experienced increased DNA damage and altered gene expression after exposure to relatively low doses of ionizing radiation [71]. Visweswaran et al. investigated DNA damage by measuring γ-H2AX foci and the expression of 12 candidate genes in the blood lymphocytes of patients exposed to low doses of X-ray radiation during neurointerventional procedures. Their study confirmed that patients undergoing these procedures received significant radiation doses, leading to DNA damage and changes in gene expression [72]. Research has demonstrated a link between radiation exposure and an increase in double-strand DNA breaks, as well as changes in DNA methylation patterns. Furthermore, the radiation dose from CT scans rises significantly with their growing clinical use. A study analyzing blood samples from healthy individuals at three intervals—before the CT scan, 1 h after, and 24 h post-scan—found altered DNA methylation one hour after the CT scan. Residual γ-H2AX foci were still present 24 h later, and the DNA methylation changes caused by the CT scan might not fully revert within that time frame. The CpG site linked to the PAX5 gene could potentially serve as a biomarker for low-dose radiation exposure [99].

Identifying individuals with genetic polymorphisms that decrease in DNA repair efficiency is important for prevention efforts, as it helps pinpoint people who can be specifically targeted to reduce cancer risks. Aka et al. conducted a multivariate analysis and found that in the exposed population, the micronuclei frequencies increased with both age and the cumulative gamma ray dose [68]. Studies evaluating the effects of long-term occupational exposure to low levels of external ionizing radiation have shown that hospital staff exposed to such radiation exhibit increased chromosomal aberrations. Engin et al. assessed whether genomic instability or changes in pteridine synthesis can serve as markers of ionizing radiation risk in hospital workers. They found that the apoptosis rates in workers exposed to gamma-radiation and X-rays were significantly higher than those in the control group. Additionally, there were notable increases in sister chromatid exchange frequency in both radiation-exposed groups. These findings suggest that prolonged exposure to low-dose ionizing radiation, even within permissible limits, can lead to increased oxidative stress, resulting in DNA damage and mutagenicity [69].

Lalic et al. discovered a higher incidence rate of structural chromosomal aberrations in peripheral blood lymphocytes of medical staff who were professionally exposed to ionizing radiation. They identified a significant positive correlation between the duration of exposure to ionizing radiation and the presence of acentric fragments. Additionally, their analysis revealed that individuals exposed to gamma-radiation exhibit the highest frequency of structural aberrations per cell. [70]. This study investigated how technogenic radiation affects the methylation levels of promoters in genes related to apoptosis in the blood lymphocytes of workers subjected to extended occupational γ-radiation exposure. Among other things, the researchers found a correlation between the level of external radiation exposure and degree of methylation of the promoters of *BAD*, *BID*, and *HRK* genes [100]. Technicians working in intensive care units regularly participate in radiological examinations to diagnose diseases. Clinicians who are occupationally exposed to X-rays at work exhibit genetic damage such as chromosomal abnormalities. Among exposed doctors from tomography departments, slightly higher percentages of micronucleus assay results and genetic damage such as chromosomal abnormalities have been observed [74]. Saini et al. investigated the impact of chronic exposure to low-dose ionizing radiation and high levels of natural radiation in individuals exposed to natural radiation. Researchers have observed a response in telomere length and the transcriptional response of telomere-specific genes and DNA damage repair genes in peripheral blood mononuclear cells [73].

## 4. Discussion

**Figure 3:**
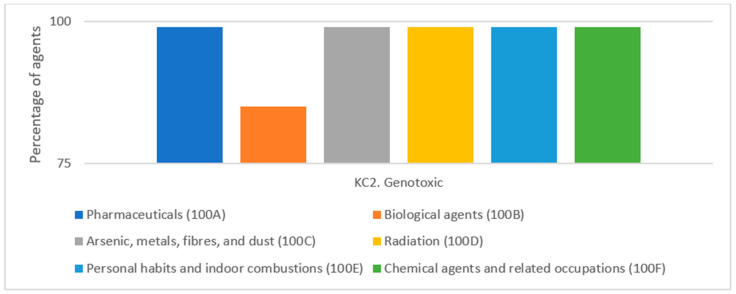
The genotoxic characteristic of group 1 86 agents by type (given as a percentage of the total number of agents). Based on Figure 6 from Krewski et al. 2019 [9].

A significant role has been attributed to the increasing use of molecular and genetic markers in research on the key characteristics of human carcinogens. Epidemiological studies conducted in vivo in humans, such as in professional environments, often analyze biomarkers reflecting DNA damage; that is, the formation of DNA adducts, clastogenic effects, and gene mutations. This makes these studies the main source of genotoxicity data. One study showed that the most prevalent mechanistic characteristic of 86 group 1 agents was genotoxic (KC2). The occurrence of genotoxicity as one of the KCs was observed in most of the 86 agents (Figure 3). Among the mechanistic characteristics, genotoxicity was the most common, identified in 85 of 86 agents. This study also showed that among the KCs for KC2, the most common source of information was human in vivo studies. Studies have been conducted in humans for the following agent types: 100A—pharmaceuticals (n = 20); 100B—biological agents (n = 10); 100C—arsenic, metals, fibers, and dust (n = 10); 100E personal habits and indoor combustions (n = 8); 100F—chemical agents and related occupations (n = 33); 100D—radiation agents (n = 5) [9].

Genotoxicity refers to the capacity to induce DNA damage, leading to the formation of DNA adducts, single- or double-strand breaks, or other alterations in chromosomal structures. This was evaluated by using three interconnected TEs: (1) DNA damage (TE1) (DNA strand breaks, DNA–protein cross-links, and unscheduled DNA synthesis), which involves changes in the chemical structure or integrity of the DNA, including strand breaks and chemical modifications such as covalent binding to nucleotide bases [101]; (2) gene mutations (TE5), indicating alterations in the normal nucleotide sequence of cellular DNA, potentially playing a crucial role in the development of human carcinogenesis [102]; (3) cytogenetic and clastogenic effects (TE7) (e.g., chromosome aberrations, micronuclei), which are harmful to chromosomes, such as DNA breakage, reorganization, acquisition, or loss of chromosome fragments [103]. An imbalance between oxidative and antioxidant activities results in oxidative stress, which may cause significant DNA damage. This damage encompasses single- and double-strand breaks, as well as modifications of DNA bases, such as the conversion of guanine to 8-hydroxyguanine. Such DNA damage acts as a trigger for genotoxic processes—including the alteration of gene transcription and activation or inactivation of critical genes—leading to mutations that contribute to aging and the development of cancer [104].

Our review presents numerous studies conducted on healthy individuals concerning occupational exposure, although not exclusively. Genotoxicity assessments have mainly been conducted on biological materials such as blood, peripheral blood lymphocytes, urine, and buccal epithelial cells. The most frequently examined genotoxic effects were DNA damage, chromosomal abnormalities, micronuclei, and sister chromatid exchange. The sister chromatid exchange assay was removed from the Organization for Economic Co-Operation and Development’s test guidelines due to insufficient clarity regarding the underlying mechanisms responsible for the effects it measures [105].

Many research groups are utilizing new opportunities in genotoxicity studies, each time grappling with the quantitative interpretation of their findings. However, these opportunities have certain limitations regarding the genotoxic effects of the selected biological material. As with genetic markers, it is difficult to extrapolate from the molecular or cellular level to the level that represents an organism’s overall response. This is due to the high variability in protective metabolic pathways, DNA repair, and combined effects on genome integrity. Therefore, there is still a lack of standardized data that definitively establish a dose–response relationship to predict the delayed occurrence of adverse health effects.

Bladder cancer ranks as the tenth most frequently diagnosed type of cancer and is one of the most frequent urinary-tract-associated malignancies worldwide. Chemical carcinogens from occupational exposure and the general environment are risk factors for bladder cancer. Moreover, exposure to occupational carcinogens is the second most common risk factor for bladder cancer in industrialized nations. Therefore, in the context of carcinogenesis, the genotoxicity of chemical agents related to environmental and occupational exposure has been extensively studied and characterized in healthy individuals.

## Figures and Tables

**Table 1:** Agents or occupational exposures with sufficient evidence in humans for bladder cancer.

Agent or Occupational Exposure	Risk Factor	Volume	Evaluation Year/Volume Publication Year	Other Cancer Sites with *Sufficient* Evidence in Humans ^a^
Aluminum production	Occupation	34, Sup 7, 92, 100F	2009/2012	ND
4-Aminobiphenyl	Occupational agents: dye and rubber manufacturing	1, Sup 7, 99, 100F	2009/2012	ND
Arsenic and inorganic arsenic compounds	Environmental factor	23, Sup 7, 100C	2009/2012	Lung, skin (malignant non-melanoma)
Auramine production	Occupation—dye industry	Sup 7, 99, 100F	2009/2012	ND
Benzidine	Occupation—dye manufacturing	29, Sup 7, 99, 100F	2009/2012	ND
Chlornaphazine	Medications or drugs	4, Sup 7, 100A	2008/2012	ND
Cyclophosphamide	Medications or drugs	26, Sup 7, 100A	2008/2012	Acute myeloid leukemia
Firefighter	Occupation	98, 132	2022/2023 online	Mesothelium (pleura, peritoneum, and other)
Magenta production	Occupation—dye industry	Sup 7, 57, 99, 100F	2009/2012	ND
2-Naphthylamine	Occupational agents: dye and rubber manufacturing	4, Sup 7, 99, 100F	2009/2012	ND
Opium consumption	Medications or drugs	126	2020/2021 online	Larynx, lung
Painter	Occupation	47, 98, 100F	2009/2012	Lung, mesothelium (pleura, peritoneum, and other)
Rubber manufacturing industry	Occupation	28, Sup 7, 100F	2009/2012	Lung, leukemia: all combined; lymphoma: all combined, stomach
*Schistosoma haematobium* infection	Disease	61, 100B	2009/2012	ND
Tobacco smoking	Smoking	83, 100E	2009/2012	Oral cavity, pharynx: all combined, oesophagus, stomach, colon, rectum, liver, bile duct, pancreas, nasal cavity and paranasal sinus, larynx, lung, upper aerodigestive tract (oral cavity, pharynx, larynx, oesophagus), uterine cervix, ovary, kidney, renal pelvis and ureter, acute myeloid leukemia, chronic myeloid leukemia
ortho-Toluidine	Occupational agents: dye and rubber manufacturing	Sup 7, 77, 99, 100F	2009/2012	ND
X- and gamma-radiation	Environmental factor	75, 100D	2009/2012	Salivary gland, oesophagus, stomach, colon, lung, bone, skin (malignant non-melanoma), breast, kidney, brain and central nervous system, thyroid, leukemia, multiple sites (unspecified)

^a^ List of classifications by cancer sites with sufficient evidence in humans, IARC Monographs Volumes 1–13; ND: no data.

**Table 2:** The systematic literature review included studies reporting genotoxic changes in healthy individuals exposed to carcinogenic agents or occupational factors with sufficient evidence linking them to bladder cancer.

Agent or Occupational Exposure	References	Years	Exposed/Control Group	Exposed Population	Genotoxic Effect on Healthy Humans	Materials
Aluminum production/aluminum exposure	Heussner et al. [15]	1985	50/50	USA aluminum reduction plant	Chromosome aberration rates	Blood samples
	Schoket et al. [16]	1993	46/29	Hungarian primary aluminum production plant workers	DNA adducts	Peripheral blood lymphocytes
	Schoket et al. [17]	1995	241/167	Hungarian primary aluminum plants workers	DNA adducts	Peripheral blood lymphocytes
	Schoket et al. [18]	1999	172/127	Hungarian aluminum plants workers	DNA adducts	Peripheral blood lymphocytes
	Carstensen et al. [19]	1999	98/55	Sweden potroom workers	Aromatic DNA adducts	Blood samples
	Carstensen et al. [20]	1999	98/55	Sweden potroom workers	DNA single-strand breaks	Blood samples
	Schoket et al. [21]	2001	161/-	Hungarian potroom workers in aluminum production	Carcinogen-DNA adducts/aromatic DNA adducts	Peripheral blood lymphocytes
	Iarmarcovai et al. [22]	2005	60/30	France welders	DNA strand breaks	Blood samples
4-Aminobiphenyl	Souza et al. [23]	2023	300/-	Brazilian pregnant women	DNA damage	Urine samples
Auramine production	ND	ND	ND	ND	ND	ND
Benzidine	Rothman et al. [24]	1996	33/15	Indian workers that manufactured benzidine dihydrochloride- and benzidine-based dyes	DNA adducts	Exfoliated urothelial cells
	Rothman et al. [25]	1997	32/15	Indian that manufactured benzidine dihydrochloride- and benzidine-based dyes	DNA adducts	Exfoliated urothelial cells
	DeMarini et al. [26]	1997	30/15	Indian that manufactured benzidine dihydrochloride- and benzidine-based dyes	DNA adducts	Urine samples
Chlornaphazine	ND	ND	ND	ND	ND	ND
Cyclophosphamide	Burgaz et al. [27]	2002	20/18	Turkish nurses, occupational exposure to antineoplastic drugs	Genetic damage	Peripheral blood lymphocytes
	Cavallo et al. [28]	2005	30/30	Italian healthcare workers regularly handling antineoplastic drugs	Micronucleus, chromosomal aberrations	Peripheral blood lymphocytes and exfoliated buccal cells
	Ursini et al. [29]	2006	30/30	Italian healthcare workers: pharmacy technicians, nurses	DNA damage	Peripheral blood lymphocytes and exfoliated buccal cells
	Rekhadevi et al. [30]	2007	60/60	Indian nurses from the oncology department	DNA damage, micronuclei	Blood samples, buccal epithelial cells
Firefighter	ND	ND	ND	ND	ND	ND
Magenta production	ND	ND	ND	ND	ND	ND
2-Naphthylamine	Souza et al. [23]	2023	300/-	Brazilian pregnant women	DNA damage	Urine samples
Opium consumption	ND	ND	ND	ND	ND	ND
Painter	Maksoud et al. [31]	2018	50/50	Egyptian painters	Genotoxic properties	Venous blood samples
	Testa et al. [32]	2005	25/37	Italian car painters	DNA damage, chromosomal aberrations, sister chromatid exchange ^1^, and micronuclei	Peripheral blood
	Pinto et al. [33]	2000	25/25	Mexican painters of public buildings	Chromosomal abnormalities, sister chromatid exchanges ^1^	Peripheral blood lymphocyte cultures
					Micronuclei	Buccal cells
	Madhavi et al. [34]	2008	102/50	Indian industrial painters	Chromosomal aberrations	Peripheral blood lymphocytes
	Siebel et al. [35]	2010	34/10	Brazilian car painters	DNA damage	Exfoliated buccal cells
	Oliveira et al. [36]	2012	58/30	Brazilin paint industry workers	DNA damage	Blood lymphocytes and oral mucosa cells
					Micronuclei	Oral mucosa cells
	Londono-Velasco et al. [37]	2016	52/52	Colombian car spray painters	DNA damage	Blood samples
	Kianmehr et al. [38]	2016	14/14	Iranian construction painters	DNA damage	Blood lymphocytes
	Pereira da Silva et al. [39]	2012	24/19	Brazilin car painters	Micronuclei	Exfoliated oral mucosa cells
					DNA damage	Peripheral blood
	Londono-Velasco et al. [40]	2019	62/62	Colombian car spray painters	DNA damage	Peripheral blood lymphocyte
	Cetintepe et al. [41]	2023	110/60	Turkish automotive paint workers	DNA damage	Peripheral blood lymphocyte
	Sisto et al. [42]	2020	17/-	Italian professional painters working in a naval industry	DNA damage	Whole venous blood samples
	KanuPriya et al. [43]	2023	30/30	Indian skilled paint workers	DNA damage, chromosomal instability or damage	Peripheral blood lymphocyte
	Hoyos-Giraldo et al. [44]	2016	150/150	Colombian car painters	Micronuclei	Exfoliated urothelial cells from voided urine
	Chang et al. [45]	2011	15/49	Taiwanese spray painters	DNA damage	Urine samples
	Cassini et al. [46]	2011	33/29	Brazilian painters from metal–mechanic industries	DNA damage	Peripheral blood and buccal cell samples
	Moro et al. [47]	2012	34/27	Brazilian industrial painters	DNA damage	Whole blood
	Villalba-Campos et al. [48]	2016	33/33	Colombian car painters	DNA damage	Peripheral blood
	Dos Reis Filho et al. [49]	2019	37/37	Brazilin car painters	DNA damage	Buccal mucosa cells
	Brum et al. [50]	2020	34/19	Brazilian painters and gasoline station attendants	DNA damage	Blood samples
	Cavallo et al. [51]	2021	17/18	Italian shipyard painters	DNA damage	Blood samples
	Varona-Uribe et al. [52]	2020	62/60	Brazilian car workshops with exposure to the organic solvents	Micronuclei	Peripheral blood lymphocyte
	Khan et al. [53]	2010	30/30	Indian painters	Micronuclei	Buccal epithelial cells
Rubber manufacturing industry	Sorsa et al. [54]	1983	55/35	Finnish rubber workers	Chromosomal aberrations, sister chromatid exchanges ^1^	Peripheral blood
	Sasiadek et al. [55]	1992	21/14	Polish vulcanizers form a tire factory	Chromosomal abnormalities, sister chromatid exchanges ^1^	Blood samples
	Sasiadek et al. [56]	1993	26/25	Polish vulcanizers form a tire factory	Sister chromatid exchanges, proliferation indices	Peripheral blood lymphocytes
	Moretti et al. [57]	1996	19/20	Italian rubber workers	DNA damage, sister chromatid exchanges ^1^ and micronuclei frequency, and proliferative rate index	Blood samples
	Major et al. [58]	1999	212/188	Hungarian rubber workers and other	Chromosomal aberration	Peripheral blood lymphocyte
	Somorovská et al. [59]	1999	29/22	Slovak rubber tire factory	DNA damage, micronucleus formation, chromosomal aberrations	Whole blood, peripheral blood lymphocyte
	Schoket et al. [18]	1999	61/76	Hungarian vulcanizing plant workers	DNA adducts	Peripheral lymphocytes
	Ward et al. [60]	2001	22/15	Texas styrene–butadiene rubber plant workers	Mutations in a reporter gene, *hprt*	Peripheral blood lymphocyte
	Ammenheuser et al. [61]	2001	31/32	Texas styrene–butadiene rubber plant workers	Mutations in a reporter gene, *hprt*	Peripheral blood lymphocyte
	Vermeulen et al. [62]	2002	52/-	Dutch rubber workers	DNA adducts and mutagenicity	Urine samples
	Vermeulen et al. [63]	2003	105/-	Dutch rubber workers	Mutagenic activity	Urine samples
	Peters et al. [64]	2008	116/-	Dutch rubber workers	DNA adducts and mutagenicity	Urine and blood samples
	Teixeira et al. [65]	2010	52/54	Portuguese workers plants manufacturing fiberglass-reinforced plastics	DNA damage, sister chromatid exchanges ^1^, micronucleus	Whole blood
	Talaska et al. [66]	2012	43/-	Dutch rubber workers	DNA adducts	Exfoliated urothelial cells
*Schistosoma haematobium* infection	Santos et al. [67]	2014	93/-	Angolan women	Catechol-estrogens/DNA adducts	Urine samples
*ortho*-Toluidine	ND	ND	ND	ND	ND	ND
X- and Gamma-radiation	Aka et al. [68]	2004	32/31	Belgian seasonal cleaners of the nuclear reactor	DNA damage, micronuclei	Whole blood, heparinized venous blood samples, peripheral blood lymphocyte
	Engin et al. [69]	2005	53/22	Radiology workers, gamma-radiation-exposed technicians, X-ray-exposed technicians	Sister chromatid exchange ^1^, DNA fragmentation	Peripheral blood lymphocyte
	Lalic et al. [70]	2005	47/20	Medical staff members exposed to gamma-radiation, X-radiation	Chromosome aberrations, acentric fragments, chromatid breaks	Peripheral blood lymphocyte
	Visweswaran et al. [71]	2019	51/-	Indian patients who underwent neuro-interventional radiological procedures	DNA damage	Peripheral blood lymphocyte
	Visweswaran et al. [72]	2020	25/-	Indian patients who underwent neuro-interventional radiological procedures	DNA damage	Peripheral blood lymphocyte
	Saini et al. [73]	2022	71/-	Indian healthy volunteers	DNA damage response	Peripheral blood mononuclear cells
	Bhotla et al. [74]	2023	100/100	Indian radiographers	Chromosomal aberrations, sister chromatid exchange ^1^, micronucleus assay	Peripheral blood lymphocyte

^1^ No longer accepted for regulatory use (removed from the Organization for Economic Co-Operation and Development Test Guidelines list); ND: no data.
